# Supplementary material for: Cross-Species Proteomics Identifies CAPG and SBP1 as Crucial Invasiveness Biomarkers in Rat and Human Malignant Mesothelioma
Source: Cancers (Basel). 2020 Aug 27;12(9):2430. doi: 10.3390/cancers12092430 (PMC7564583; doi:10.3390/cancers12092430)
Supplement: Supplementary file 1 [file cancers-12-02430-s001.docx]

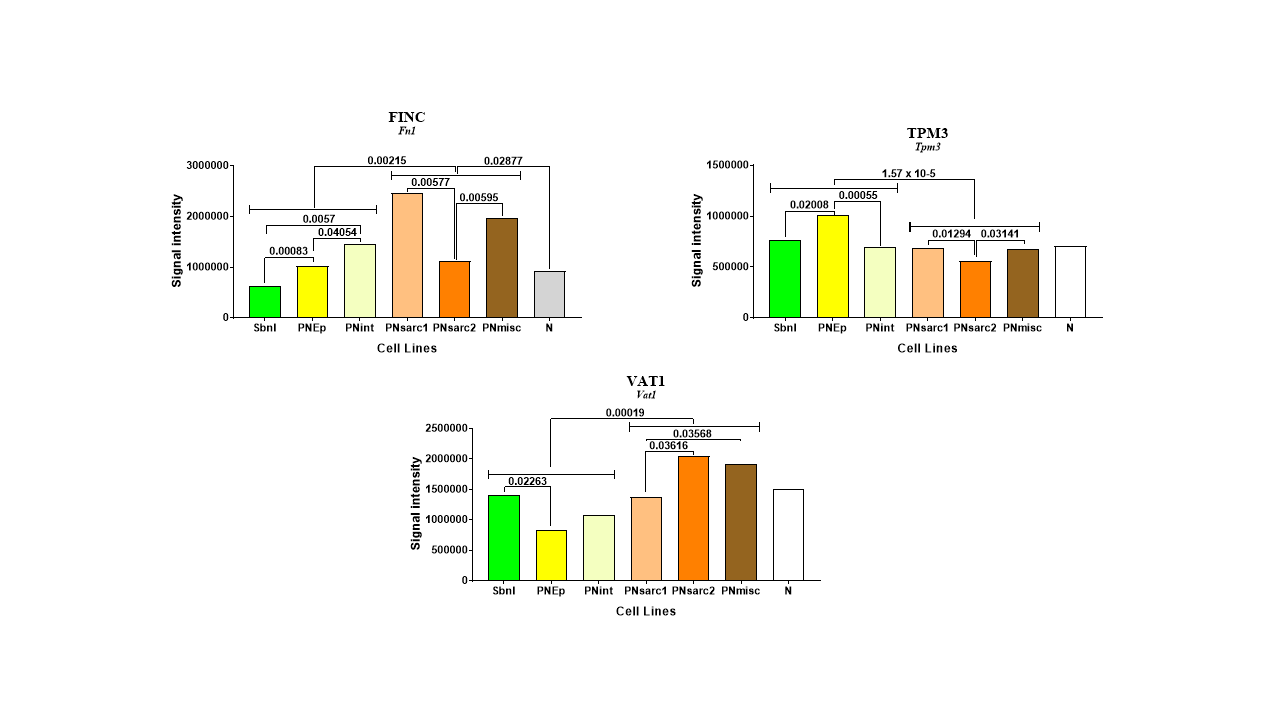


**Figure S1:** Additional biomarkers of human vs. rat MM and rat mesothelial cell carcinogenesis. For fibronectin (**A**), a progressive rise in abundance was observed starting within the different subgroups of preneoplastic cell lines with epithelioid morphology, and continuing from PNint to PNsarc1. (**B**), and (**C**), Evolution of abundance changes for TPM3 and VAT1, respectively.
